# Special Issue Editorial: Advanced Science for Tissue‐Interfaced Organic Bioelectronics

**DOI:** 10.1002/advs.78375

**Published:** 2026-10-22

**Authors:** Tae‐il Kim, Shiming Zhang, Simone Fabiano

**Affiliations:** ^1^ School of Chemical Engineering Sungkyunkwan University (SKKU) Suwon Republic of Korea; ^2^ Department of Electrical and Computer Engineering The University of Hong Kong Hong Kong SAR China; ^3^ School of Biomedical Engineering The University of Hong Kong Hong Kong SAR China; ^4^ Laboratory of Organic Electronics Department of Science and Technology Linköping University Norrköping Sweden

The interface between living tissues and electronic systems represents one of the most important frontiers in modern bioelectronics. Biological systems communicate through complex combinations of electrical, ionic, chemical, and mechanical signals, whereas conventional electronics predominantly operate through electronic charge transport in rigid materials. Bridging these fundamentally different domains therefore requires more than simply miniaturizing conventional electronic devices. It requires materials and devices that can establish intimate, stable, and ultimately functional interactions with all living systems, including cells, tissue, and the body.

Organic electronic materials are particularly well positioned to address this challenge. Their mechanical compliance, biocompatibility, and ability to support molecular tunability for mixed ionic–electronic transport provide unique opportunities to communicate with biological systems in ways that are difficult to achieve using conventional inorganic electronics. The development of conducting polymers, organic mixed ionic–electronic conductors (OMIECs), organic electrochemical transistors (OECTs), hydrogels, and ionically active materials has consequently transformed bioelectronics from a field largely concerned with recording electrical signals into one capable of sensing, processing, and modulating diverse biological activities. The 24 contributions collected in this Special Collection—comprising 16 Research Articles, 7 Reviews, and 1 Perspective—capture this transition and illustrate the rapidly expanding scope of tissue‐interfaced organic bioelectronics [1, 2, 3, 4, 5, 6, 7, 8, 9, 10, 11, 12, 13, 14, 15, 16, 17, 18, 19, 20, 21, 22, 23, 24].

A central theme emerging from this collection is the increasing importance of the interface itself as a functional component of the bioelectronic system. Yamagishi et al. review soft, flexible, and stretchable platforms capable of establishing mechanically compliant interfaces with dynamic biological tissues, including skin, neural tissue, and wet organs [1]. Park et al. extend this concept toward multifunctional adaptive interfaces that can respond to mechanical and physiological environments through shape programmability, self‐healing, permeability, tissue adhesion, biodegradability, and strategies for reducing adverse biological responses [2]. Boahen et al. further highlight autonomous polymer frameworks that introduce self‐adhesion, self‐healing, self‐protection, self‐sensing, and self‐degradation into sustainable wearable and implantable bioelectronics [3]. Together, these contributions emphasize that successful tissue integration requires not simply mechanical softness, but an ability to continuously accommodate complex and changing biological environments.

At the materials level, PEDOT:PSS and other organic mixed ionic–electronic conductors continue to form a central foundation of organic bioelectronics. Ávila Ramírez et al. provide a Perspective on PEDOT:PSS, highlighting its tunable electronic and ionic conductivity, processing versatility, and widespread use in physiological sensing and stimulation [4]. Ye et al. discuss ionic liquids as artificial ionic materials capable of bridging biological ion‐based signaling with electron‐based electronic devices and describe their emerging roles in sensing, actuation, transport, and adaptive bioelectronic functions [5]. Moving from molecular design to 3D architectures, Kim et al. demonstrate arbitrary 3D OMIEC structures through the controlled self‐fusion of PEDOT:PSS microfibers [6]. Sun et al. introduce antibacterial and biodegradable ε‐poly‐L‐lysine‐graft‐oligo(3‐hexylthiophene) copolymers, illustrating how electronic functionality can be combined with transient and biologically favorable material properties [7]. These contributions demonstrate how chemistry, processing, and structural design are collectively expanding organic bioelectronic materials beyond conventional planar conducting films.

OECTs constitute another major theme of the Special Collection because of their unique capability to couple ionic signals in biological environments with electronic amplification. Bai et al. review the fundamental operating principles, material design, and chemical, epidermal, and implantable interfaces of OECTs [8]. Travaglini et al. examine the emerging integration of OECTs into logic circuits, highlighting a transition from simple biological signal transduction toward local information processing at the biointerface [9]. Chang et al. demonstrate a time‐multiplexed, nanobody‐functionalized OECT platform for rapid multiplexed detection of respiratory viral proteins in saliva [10]. Liang et al. develop all‐hydrogel n‐type OECTs that combine efficient mixed conduction with tissue‐like mechanical properties for epidermal and implantable physiological monitoring [11]. Hu et al. introduce dynamically tunable OECT‐based inverter circuits for stable in situ electrophysiological monitoring [12], while Han et al. extend ion‐selective OECT sensing to continuous in vivo potassium monitoring in the living plants [13].

Importantly, several contributions demonstrate that conventional electronic figures of merit alone cannot fully describe the performance of tissue‐interfaced devices. Zemignani et al. show that high transconductance does not necessarily ensure high‐fidelity cellular electrophysiological recordings; instead, cell–polymer coupling critically determines whether cellular action potentials are effectively transduced [14]. This study captures an important conceptual shift for the field: the performance of tissue‐interfaced bioelectronics should increasingly be evaluated as a property of the integrated material–device–tissue system, rather than of the electronic component in isolation. In a complementary biomimetic approach, Pavlou et al. develop a methodology for deciphering resistance variability in supported lipid bilayers, demonstrating how membrane organization and defects can influence electrical measurements across biological model interfaces [15].

A further major direction represented in this collection is the translation of organic and soft materials toward physiological sensing and clinically relevant monitoring. Hydrogel‐based electrodes feature prominently because they provide hydrated, compliant, and conformable interfaces with tissues. Cui et al. develop synthetic hydrogel electrodes to improve the skin conformability of wearable continuous glucose monitors [16]. Alsaafeen et al. systematically benchmark conductive hydrogel electrodes for noise‐tolerant epidermal bioelectronics, addressing an important practical challenge in real‐world electrophysiological monitoring [17]. Marchianò et al. take soft bioelectronic sensing into the gastrointestinal environment by developing edible Ca^2^^+^‐crosslinked alginate hydrogel electrodes for glucose monitoring [18]. These studies collectively illustrate how interface materials can be tailored for physiological environments ranging from the epidermis to the gastrointestinal tract.

The collection also highlights increasingly sophisticated implantable and tissue‐integrated systems. Choi et al. develop shape‐morphable, biodegradable, and brain‐adhesive bioelectronics capable of conforming to cortical surfaces for stable brain‐signal monitoring [19]. Wahl et al. demonstrate implantable resorbable organic impedance sensors for real‐time in vivo monitoring of intestinal ischemia, illustrating the clinical potential of transient electronic systems that do not require permanent implantation or subsequent removal [20]. Wheeler et al. develop a conformable device that independently monitors mucosal and vascular barriers in a complex intestinal model, demonstrating how tissue‐interfaced electronics can also improve the physiological relevance of advanced in vitro systems [21]. Together, these contributions reflect a broader shift from proof‐of‐concept devices toward platforms designed around specific biological and medical requirements.

Beyond sensing and monitoring, tissue‐interfaced organic materials are increasingly becoming active modulators of biological function. Marzuoli et al. use semiconducting polymer nanoparticles to achieve light‐controlled bidirectional modulation of nitric oxide in endothelial cells, illustrating how organic materials can directly regulate cellular signaling pathways [22]. Ondráčková et al. investigate the electrochemical mechanisms underlying direct‐current nerve lesioning and establish an electrochemical neuromodulation modality termed the “Faraday Scalpel.” [23]. More broadly, Ro et al. review ionic–bionic interfaces in which mobile ions serve not only as biological signal carriers but also as functional mediators for sensing, wound treatment, responsive drug delivery, and therapeutic intervention [24]. These studies demonstrate an important progression from passive observation toward bidirectionally active interaction with living systems.

Taken together, the articles in this Special Collection reveal several important emerging directions. First, mechanical compliance is evolving toward adaptive integration, in which biointerfaces accommodate motion, deformation, swelling, healing, degradation, and changing physiological environments. Second, mixed ionic–electronic transport is enabling closer correspondence between the operation of electronic devices and the intrinsically ionic nature of biological signaling. Third, bioelectronic systems are progressing from passive recording toward integrated sensing, local signal processing, stimulation, and therapeutic intervention. Finally, the increasing emphasis on biodegradable, resorbable, edible, and sustainable materials reflects a growing recognition that next‐generation bioelectronics must consider not only device performance but also their complete biological and environmental life cycle.

Considerable challenges nevertheless remain. Long‐term stability, tissue adhesion, biofouling, foreign‐body responses, molecular selectivity, device‐to‐device variability, scalable manufacturing, power delivery, data communication, and validation under clinically relevant conditions must all be addressed. Equally important will be the development of quantitative metrics that characterize the quality of the biological interface rather than electronic performance alone. Future advances will therefore require increasingly close integration of materials science, electronics, chemistry, biology, medicine, and data science. The next frontier of organic bioelectronics may thus lie not simply in making electronics softer or more flexible, but in making the tissue–electronic interface itself intelligent and functional. Such interfaces should ultimately be capable of sensing biological states, adapting to changing environments, processing information, and delivering appropriate responses while maintaining stable communication with living systems. The contributions collected in this Special Collection provide a timely snapshot of this rapidly evolving field and demonstrate how advances in organic materials, device architectures, and biological interfaces are collectively bringing us closer to seamless and bidirectional communication between electronics and living systems.

We sincerely thank all authors for their outstanding contributions to this Special Collection and the reviewers for their thoughtful and constructive evaluations. We also gratefully acknowledge the editorial team of Advanced Science for their continuous support throughout the preparation of this collection. We hope that this Special Collection will stimulate further interdisciplinary research and inspire new approaches toward next‐generation tissue‐interfaced organic bioelectronics.

## Author Contributions

**Tae‐il Kim**: Writing – original draft. **Shiming Zhang**: writing – review and editing. **Simone Fabiano**: writing – review and editing.

## Conflicts of Interest

The authors declare no conflicts of interest.

## Data Availability

Data sharing not applicable to this article as no datasets were generated or analyzed during the current study
